# Development of Large Language Model Specialized into Microbiome Datasets: an Application of Self-Evaluation and Scoring Comparison with Conventional Natural Language Processing Markers

**DOI:** 10.4014/jmb.2511.11050

**Published:** 2026-01-26

**Authors:** Chan Kyu Park, Sung Hwan Bae, Hyeon Woo Park, Nam Su Oh, Young Jun Kim, Young-Wan Kim, Tae Jin Cho, Ying Li, Jianmin Chai, Jiangchao Zhao, Hyung Taek Cho, Ji Hoon Jung, Jinbong Park, Tae Gyun Kim, Jae Kyeom Kim

**Affiliations:** 1 Department of Food and Biotechnology, Korea University, Sejong 30019, Republic of Korea; 2 Guangdong Provincial Key Laboratory of Animal Molecular Design and Precise Breeding, Foshan University, Foshan 528225, P.R. China; 3 Guangdong Laboratory for Lingnan Modern Agriculture, South China Agricultural University, Guangzhou 510642, P. R. China; 4 The Bioinformatix, Gwangmyeong 14348, Republic of Korea; 5 College of Korean Medicine, Kyung Hee University, Seoul 02447, Republic of Korea; 6 Department of Health Behavior and Nutrition Sciences, University of Delaware, Newark, DE 19711, USA

**Keywords:** Bioinformatics, Domain adaptation, Human evaluation, Large language model, Microbiome, Phi-4 metric

## Abstract

The gut microbiome plays a fundamental role in host metabolism, immune regulation, and disease development. With the rapid accumulation of multi-omics and literature data, the microbiome field now faces the challenge of efficiently extracting scientific insights from massive, heterogeneous datasets. Artificial intelligence (AI) and large language models (LLMs) provide promising tools to address this complexity by enabling integrative analysis and knowledge synthesis across diverse biological sources. In this study, we developed METABOLISM, a microbiome-specialized LLM fine-tuned on 160,000 scientific abstracts to enhance literature-based contextual understanding of microbiome–liver interactions and related biological mechanisms. Using LoRA-based parameter-efficient training, METABOLISM was optimized for domain-specific reasoning and response generation. Model performance was evaluated through both automated Phi-4 scoring (a large language model–based evaluator for relevance, informativeness, and fluency) and structured human expert rubric assessments involving 20 domain specialists. The fine-tuned METABOLISM achieved superior relevance and clarity scores (mean > 7.5 ± 0.06) compared with general-purpose LLMs such as Gemma-3-12B-IT and ChatGPT-4o. Correlation analysis revealed weak to moderate negative relationships (R = –0.65, *p* < 0.0001) between traditional NLP metrics (BLEU, ROUGE) and human expert rubric scores, with a similar trend observed for correlations with Phi-4–based automated evaluation scores, indicating the limitations of surface-level similarity measures in biomedical contexts. Overall, our findings demonstrate that microbiome-adapted LLMs can effectively distill high-volume scientific data into biologically meaningful insights, supporting more efficient and interpretable research in microbiology and systems biology.

## Introduction

The rapid advancement of artificial intelligence (AI) and large language models (LLMs) has revolutionized various fields, including life sciences, bioinformatics, and medical research (3). LLMs, powered by Transformer-based neural networks and self-attention mechanisms, have demonstrated remarkable capabilities in natural language processing (NLP), enabling efficient text analysis, summarization, and generation (14). Beyond traditional text processing, these models are increasingly being utilized for analyzing complex biological data, such as genome sequences, protein structures, and disease pathways (5). Given their ability to recognize intricate patterns and relationships, LLMs offer powerful tools for tackling biological challenges and accelerating discoveries in molecular biology and healthcare (Sarwal *et al*., 2023).

One particularly significant area where AI-driven models can provide valuable insights is the microbiome and gut-liver axis, a bidirectional communication pathway between the gastrointestinal tract and the liver (13). This axis, mediated by the portal vein, facilitates the direct transfer of gut-derived substances into the liver, influencing hepatic metabolism and immune responses (1). The gut microbiome plays a critical role in maintaining metabolic and immune homeostasis, and its dysbiosis—an imbalance in microbial composition—has been implicated in various liver diseases, including non-alcoholic fatty liver disease, alcoholic liver disease, hepatitis, cirrhosis, and liver cancer (Reuter and Bajaj, 2020). Recent studies suggest that gut microbial products, such as lipopolysaccharides and short-chain fatty acids, can trigger hepatic inflammation and fibrosis, emphasizing the need for a deeper understanding of microbiome-liver interactions (Lin *et al*., 2022).

Despite the growing body of research on the gut-liver axis, analyzing and interpreting microbiome-related data remain significant challenges due to the complexity of microbial ecosystems and their dynamic interactions with host physiology (Gerussi *et al*., 2022). While existing LLM-based biological analysis models focus primarily on structured microbiome sequencing data or unstructured biomedical literature, they often lack the ability to comprehensively integrate both data types (Yu *et al*., 2023). This limitation hinders the generation of precise and context-aware insights into microbiome-liver associations (Taha *et al*., 2022).

To bridge this gap, we introduce METABOLISM, a novel LLM framework specifically designed to analyze microbiome-liver interactions by integrating microbiome-related literature to support biologically grounded reasoning. To this end, this study aims to systematically evaluate our models’ performance in comparison to existing LLMs for microbiome-liver relationship analysis. Specifically, we assess METABOLISM’s ability to provide a comprehensive analysis of microbiome-liver interactions, support literature-grounded discussion of disease-associated mechanisms by identifying microbiome patterns linked to liver disease progression, and enhance interpretability in microbiome-driven liver disease studies, enabling more precise hypothesis generation and experimental design (Cheungpasitporn *et al*., 2024). By benchmarking METABOLISM our model against other models, this study highlights its advantages in handling complex biological datasets and advancing AI-driven research methodologies in microbiome-liver interactions. Furthermore, it has the potential to extend beyond literature analysis, contributing to disease prediction, diagnosis, and the development of personalized therapeutic strategies in the future. Rather than aiming to derive or validate novel biological mechanisms, the primary objective of this study is to evaluate whether a microbiome-specialized LLM can support literature-based understanding and interpretation of microbiome–liver axis research through domain-adapted language modeling.

## Materials and Methods

### Data Collection and Preprocessing

A large-scale microbiome text corpus was constructed to develop and fine-tune the METABOLISM model. In total, 160,000 titles and abstracts of microbiome-related research articles published up to December 2024 were retrieved exclusively from publicly available sources, namely the PubMed and Europe PMC databases. Keyword queries including “microbiome,” “gut microbiota,” “metagenomics,” “intestinal flora,” “liver,” and “host–microbe interaction” were used to collect literature relevant to microbiome–liver axis research. Based on keyword co-occurrence analysis, approximately 30% of the collected articles explicitly focused on liver-related topics (*e.g.*, liver, hepatic metabolism, NAFLD, cirrhosis), while the remaining articles covered broader microbiome research. Thus, the corpus represents an enriched, but not exclusive, microbiome–liver axis–focused dataset. Duplicated or incomplete entries and non-English records were excluded. All text data were processed using Python (v3.11) and the Hugging Face Datasets library. Preprocessing involved: (i) lowercasing all characters, (ii) removing special characters and punctuation, (iii) tokenization using the Gemma-3-12B-IT model’s native tokenizer, and (iv) truncation to a maximum input length of 4,096 tokens per entry. To minimize information loss in biomedical expressions, preprocessing was applied conservatively; alphanumeric tokens, hyphenated terms, and entity-defining symbols commonly used in biomedical texts (*e.g.*, cytokine names, microbial strain identifiers, and statistical notations) were preserved, while normalization was limited to non-informative punctuation and formatting artifacts. The preprocessed dataset was randomly shuffled to minimize ordering bias and subsequently divided into training (90%) and validation (10%) subsets to ensure balanced representation across topics and publication years.

### Computing Environment and Software Framework

The experiments were carried out in a computing environment running Ubuntu 24.04.2 LTS as the operating system. Model training and fine-tuning were implemented using the Unsloth framework, known for its high-performance capabilities, in conjunction with the Hugging Face Transformers library. To enhance computational efficiency, bfloat16 mixed precision was utilized throughout training. Furthermore, FlashAttention v2 was employed to accelerate attention mechanisms, and Optuna was integrated for hyperparameter optimization.

### Model Architecture

The base model used in this study was Gemma-3-12B-IT, a multimodal instruction-tuned model comprising separate text and vision components. All experiments were conducted in a text-only configuration, and no visual inputs were provided during training or inference. Accordingly, parameters associated with the vision encoder were frozen and excluded from optimization during fine-tuning. The model was configured to accept a maximum input length of 4,096 tokens, enabling it to handle long-form textual data effectively. To preserve pre-trained visual representations and reduce computational overhead, vision-related layers were frozen during fine-tuning. The trainable parameters were limited to specific language components, including: Transformer language layers; Attention modules (q_proj, k_proj, v_proj, o_proj, gate_proj); Multi-layer perception modules (up_proj, down_proj); and both the embedding layer (embed_tokens) and output head (lm_head). This selective training strategy aimed to optimize performance while minimizing training time and resource usage.

### Model Fine-Tuning

To enhance efficiency during fine-tuning, we applied a combination of optimization and memory-saving techniques. Low-rank adaptation (LoRA) was employed for parameter-efficient tuning, enabling effective adaptation of the model with significantly fewer trainable parameters. To further reduce GPU memory consumption, gradient checkpointing was utilized. For computational acceleration, FlashAttention v2 was integrated to speed up attention operations. The training process adopted the adamw_torch_fused optimizer, which combines the AdamW update rule with the compute unified device architecture (also known as CUDA)-level optimization for faster performance. All computations were performed using bfloat16 precision to balance numerical stability and hardware efficiency. In the present study, METABOLISM was trained using a single-stage domain-adaptive fine-tuning procedure on a fixed microbiome literature corpus. Domain-Adaptive Continual Fine-Tuning (DACFT) was not implemented here and is described solely as a future extension to support incremental updates as new literature becomes available.

### Hyperparameter Optimization

Hyperparameters were optimized using the Optuna framework with a Tree-structured Parzen Estimator (TPE) sampler. The following parameters were tuned: Learning rate (lr); Number of epochs; Batch size; Gradient accumulation steps; LoRA configuration (rank r, scaling factor α, dropout rate); Weight decay; Warmup ratio; and Optimizer type. After iterative sampling and evaluation, the final selected hyperparameters were as follows: lr = 8.92 × 10^-5^; epochs = 8; batch_size = 1; accum_steps = 1; and LoRA: r = 16, α = 32, dropout = 0.1.

### Phi-4 Based Evaluation Protocol

To evaluate the performance of the fine-tuned model, we randomly selected article titles from the original dataset. Each title was provided as input to the model, which then generated a summary based on the inferred abstract content. The Phi-4 model (fp16 version) was used as an automated evaluator to assess the quality of each generated summary. Automated evaluation was performed using Phi-4, which scored each model-generated response on relevance, informativeness, and fluency on a 0–10 scale. Evaluation was conducted using a fixed prompt with deterministic decoding (temperature = 0.0).

### Human Expert Evaluation

To evaluate the quality of responses generated by our domain-specialized METABOLISM focused on the gut-liver axis knowledge, we implemented a structured human expert assessment protocol such as QUEST (Tam *et al*., 2024) A curated set of 3 prompts was designed to comprehensively cover biological and clinical topics related to the microbiome–liver axis, gut-derived metabolites, and host–microbiome interactions. All responses were generated in isolated sessions to avoid memory bias, anonymized, and randomly ordered to eliminate evaluator bias.

A panel of 20 domain experts independently evaluated all 3 responses. Experts held PhDs or equivalent clinical/research experience (≥5 years) in microbiology, biotechnology, nutrition science, or systems biology. Each expert was blinded to the model identity and assessed responses using a five-point Likert scale across 14 dimensions: accuracy, domain relevance, clarity, usefulness, safety/harm avoidance, and structural coherence. The rubric and definitions for each dimension were aligned with the QUEST framework and are detailed in Supplementary Table 1. Inter-rater reliability across the 20 experts was assessed using intraclass correlation coefficients (ICC), demonstrating moderate to high agreement across the evaluated dimensions.

### Statistical Analysis

To test if the mean Phi-4 scores differed significantly across LLMs, a one-way ANOVA was executed followed by post-hoc Tukey HSD test (GraphPad, USA). Assumptions of normality and homogeneity of variance were verified prior to analysis. To assess the relationship between automated text similarity metrics and expert-derived Phi-4 scores, we performed a Pearson correlation analysis. Correlation analyses were conducted separately to assess associations between NLP similarity metrics (BLEU/ROUGE) and (i) human expert rubric scores, and (ii) Phi-4–derived automated evaluation scores. Pearson correlation coefficients were computed for each comparison. Lastly, to compare human expert Likert ratings across LLMs, we conducted a one-way ANOVA for each evaluation dimension (*e.g.*, accuracy, clarity). Inter-rater reliability among the 20 experts was assessed using intraclass correlation coefficients (ICC) to quantify consistency across expert ratings. Ratings were treated as interval-scale data consistent with common practices in Likert-based evaluation research. Assumptions of normality and homogeneity of variance were verified; Kruskal–Wallis tests were additionally performed as a robustness check for non-parametric distributions.

## Results and Discussion

### System Architecture of the METABOLISM Framework

**Fig. 1:**
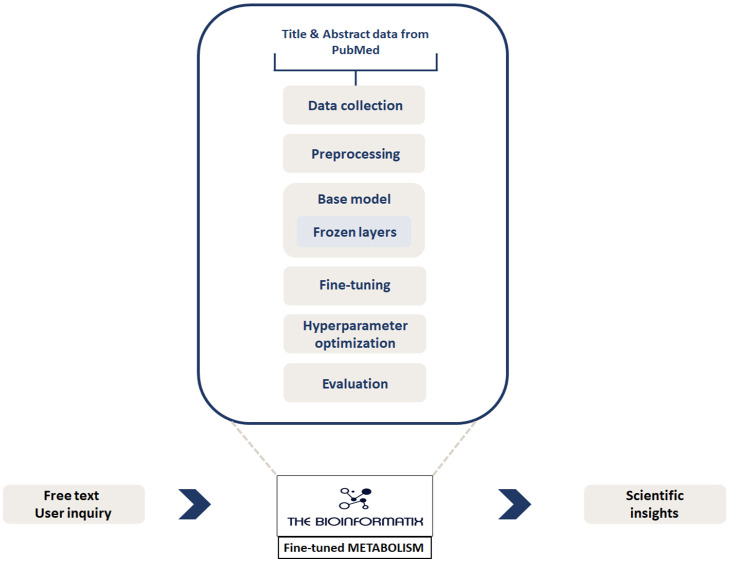
System architecture of the METABOLISM framework.

The METABOLISM framework was designed to integrate diverse microbiome-related data streams and optimize LLM performance for domain-specific applications. Fig. 1 illustrates the end-to-end workflow, which includes (i) ingestion and preprocessing of microbiome literature data, (ii) Stage-A hyperparameter optimization, (iii) LoRA-based fine-tuning of the Gemma-3-12B-IT model, and (iv) a planned domain-adaptive continual fine-tuning (DACFT) strategy (Fig. 1). The framework leverages structured knowledge graph inputs and extended SPARQL queries to iteratively refine model outputs, culminating in automated performance evaluation using the Phi-4 evaluator.

**Fig. 2:**
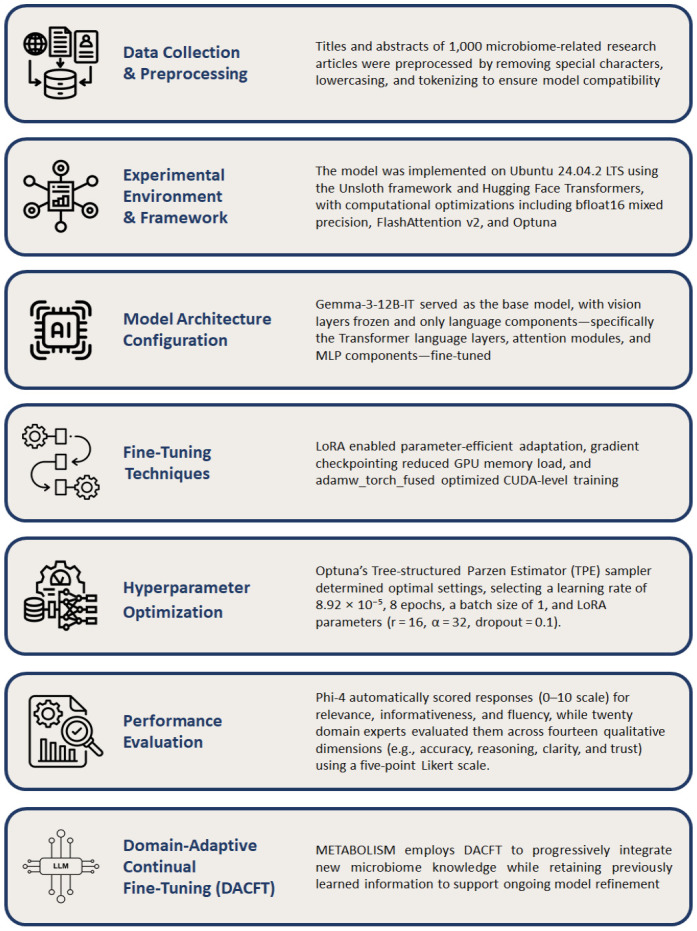
Overview of the METABOLISM workflow from data collection to domain-adaptive continual fine-tuning.

To provide a more detailed overview of the model workflow, Fig. 2 summarizes the entire METABOLISM framework, which was designed to systematically integrate microbiome literature data into a domain-specialized large language model (Fig. 2). This pipeline represents the core structure supporting METABOLISM’s ability to learn context-rich biological knowledge while maintaining computational efficiency. The framework begins with large-scale data collection and preprocessing, where microbiome-related titles and abstracts retrieved from PubMed and Europe PMC were cleaned, lowercased, and tokenized to ensure uniformity and compatibility with the model tokenizer. This curated corpus enabled comprehensive coverage of microbial community dynamics, metabolite functions, and host–microbe interactions. Subsequent stages included environment setup and LoRA-based fine-tuning within the Unsloth–Hugging Face ecosystem, followed by hyperparameter optimization using Optuna’s TPE sampler. The optimal configuration (learning rate = 8.92 × 10^-5^; 8 epochs; LoRA r = 16, α = 32; dropout = 0.1) achieved stable convergence and efficient resource utilization. Model performance was assessed through both Phi-4 automated scoring and structured human expert rubric evaluation. The moderate-to-strong consistency between automated Phi-4–based scores and human expert evaluations confirmed that METABOLISM effectively captured biologically relevant context. Phi-4 scores showed moderate-to-strong agreement with human rubric scores (Spearman ρ = 0.62; ICC = 0.71), indicating consistent evaluation trends between automated and expert-derived assessments. Finally, the DACFT module allows incremental incorporation of newly emerging microbiome knowledge, supporting ongoing refinement and adaptability of the model for future microbiome research applications.

### Performance of Fine-Tuned METABOLISM Compared to Baseline LLMs

**Fig. 3:**
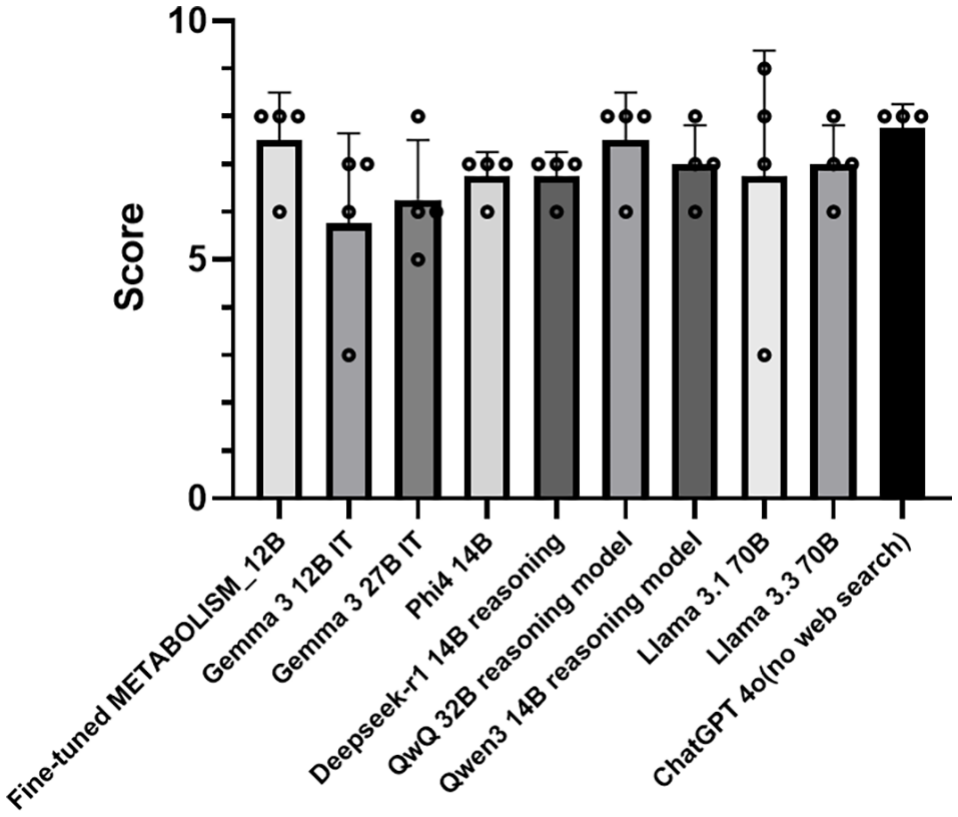
Performance comparison of the fine-tuned METABOLISM against various LLMs. The bar graph presents the mean performance scores (± standard error) of the METABOLISM fine-tuned with LoRA compared to multiple baseline LLMs, including Gemma-3-12B-IT, LLaMA-3, Phi-1.4B, Qwen, and ChatGPT-4o (no web search). Scores were derived from Phi-4 automated evaluation across microbiome-related prompts, with higher values indicating better response quality.

To assess the effectiveness of the METABOLISM model, we compared its performance against a selection of state-of-the-art LLMs, including Gemma-3-12B-IT, LLaMA-3, Phi-1.4B, Qwen, and ChatGPT-4o (no web search). As shown, the fine-tuned METABOLISM Model_12B achieved the highest mean score among all tested models, outperforming even the strongest general-purpose LLMs (Fig. 3). The METABOLISM model exhibited a consistent performance advantage, with an average score above 7.5 (± 0.06), compared to baseline models that generally scored in the 6.0-7.2 range. These findings demonstrate that targeted fine-tuning and domain adaptation substantially improved the model’s ability to process microbiome-related content. We note that this comparison is inherently influenced by domain specialization, as METABOLISM was explicitly fine-tuned on microbiome-related literature. Accordingly, these results should be interpreted as illustrating the effect of domain adaptation under a consistent evaluation setting, rather than as evidence of absolute model superiority.

### Correlation between Phi-4 Evaluation Scores and Standard NLP Metrics

**Fig. 4:**
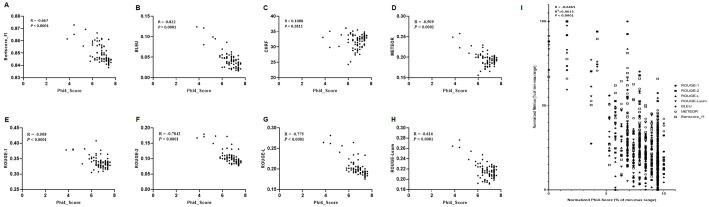
Correlation between Phi-4 evaluation scores and standard NLP metrics. Scatter plots illustrate the relationship between Phi-4 automated evaluation scores and commonly used NLP metrics, including BLEU, ROUGE-1/2/L, ROUGE-Lsum, METEOR, BERTScore, and CHRF. Overall, Phi-4 scores show weak to moderate negative correlations with most surface-level similarity metrics particularly BLEU (panel **B**) and ROUGE variants (panels **E-H**) indicating that higher n-gram overlap does not necessarily correspond to contextually accurate or useful outputs in biomedical contexts. In contrast, BERTScore (panel **A**) exhibits a less pronounced negative trend, suggesting that semantic similarity measures align somewhat more closely with human-like evaluations than purely n-gram-based metrics. The Pearson correlation coefficient (R = -0.418), the coefficient of determination (R^2^ = 0.4615), and the significance (*P* < 0.0001) summarize and confirm the overall negative correlation between Phi-4 scores and traditional n-gram and embedding-based metrics.

To better understand the relationship between human-aligned automated evaluation (*i.e.*, Phi-4 scores) and widely used NLP metrics, we examined correlations across representative metrics, including BLEU, ROUGE-1/2/L, ROUGE-Lsum, METEOR, BERTScore, and ChrF (Fig. 4). The Phi-4 scores displayed weak to moderate negative correlations with most surface-level similarity metrics, particularly BLEU (Fig. 4B) and ROUGE variants (Fig. 4E-4H; R values ranging from -0.50 to -0.82, all *p* < 0.0001). For example, models with high BLEU scores often exhibited lower Phi-4 scores, suggesting that reference-based n-gram overlap may inadequately reflect response quality when the reference abstracts differ structurally or stylistically from ideal explanatory outputs in a biomedical setting, consistent with prior reports on the limitations of BLEU and ROUGE. Conversely, BERTScore (Fig. 4A) showed a less pronounced negative trend (R = -0.667, *p* < 0.0001), implying that semantic similarity measures may align slightly better with human-like evaluations than n-gram–based metrics.

After, in order to combine the NLP metrics, the values were normalized and then applied into a linear regression (Fig. 4I). As shown, our analysis reveals a significant negative correlation between normalized Phi-4 human evaluation scores and traditional NLP evaluation metrics such as BLEU, ROUGE, METEOR, and BERTScore-F1 (R = -0.6465, *p* < 0.0001), suggesting that higher scores on conventional automated metrics are often associated with lower ratings from domain experts. This counterintuitive pattern underscores a critical misalignment between n-gram overlap-based metrics and the nuanced, context-aware criteria emphasized in human expert evaluation. In the context of biomedical question answering and microbiome-specific reasoning, such misalignment may stem from the inability of token-based metrics to capture factual accuracy, interpretative depth, and clinical coherence—dimensions that are central to expert judgment. Consequently, these findings highlight the limitations of relying solely on traditional NLP metrics to evaluate domain-specialized LLMs, and affirm the necessity of incorporating human-centric, rubric-guided assessment frameworks in high-stakes fields like biomedical research. Importantly, this study does not aim to generate or validate novel biological findings; rather, it demonstrates how a domain-adapted LLM can organize and interpret existing microbiome–liver axis knowledge from the literature, with experimental validation reserved for future work. Accordingly, the negative correlations observed in Fig. 4 should be interpreted with caution, as they primarily reflect well-documented limitations of reference-based similarity metrics, rather than inverse model quality or diminished biological relevance. These findings therefore represent a domain-specific reconfirmation of existing observations reported in prior biomedical and NLP studies, rather than a novel methodological contribution. In addition, the effects of alternative preprocessing strategies, such as relaxed normalization or entity-aware tokenization, were not systematically evaluated and represent an important direction for future work. Accordingly, applications such as hypothesis generation, disease association exploration, or experimental planning should be interpreted as literature-assisted reasoning support, with downstream biological validation required prior to predictive or translational use. Although this study emphasizes the microbiome–liver axis as a focused evaluation domain, the underlying training corpus encompasses a broad range of microbiome-related literature, including studies on host–microbe interactions beyond hepatic contexts. Therefore, METABOLISM is not inherently limited to liver-focused applications; however, systematic evaluation across other microbiome subdomains, such as the gut–brain axis or skin microbiome, remains an important direction for future work.

In addition, several limitations of the present study should be acknowledged. First, this work does not provide independent experimental or data-driven biological validation and therefore does not claim to generate or confirm novel biological mechanisms. Second, the evaluation relies primarily on expert-based qualitative assessment of generated text, which reflects biological plausibility and literature consistency rather than experimentally verified biological accuracy. Third, the evaluation design is intentionally narrow and exploratory, focusing on a limited number of prompts, and may not capture the full diversity of microbiome-related research contexts. Accordingly, the results should be interpreted as a methodological validation of a domain-adapted language model for literature-based reasoning support, with broader benchmarking and biological validation left for future work.

This phenomenon has been observed in previous studies. For example, a recent report from Hijazi *et al*. demonstrated that BLEU and ROUGE scores exhibited low correlation with human expert ratings when evaluating biomedical query-focused summarization systems, reinforcing the argument that lexical overlap is a poor proxy for content fidelity in specialized domains (Hijazi *et al*., 2024) Similarly, Sreekar *et al*. emphasized the weak correlation between traditional metrics and human judgment across summarization and free-text generation, advocating for explainable, LLM-based metrics instead (P Aditya Sreekar, 2024). Together, these evaluation results validate the model’s capacity to generate literature-consistent and biologically plausible explanations, thereby supporting its intended role in facilitating biological understanding through text-based synthesis rather than biological discovery.

### Human Expert Evaluation of Overall Response Quality

**Fig. 5:**
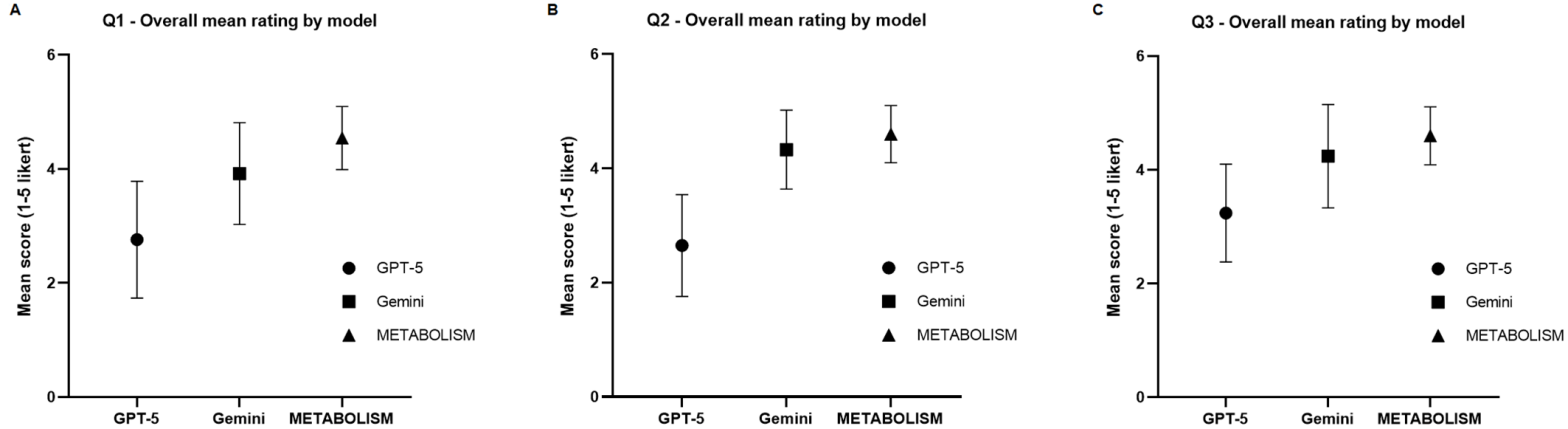
Human expert evaluation of overall response quality across LLMs. Mean expert ratings (1-5 Likert scale) for three large language models (GPT-5, Gemini, and METABOLISM) across three evaluation sets (Q1-Q3). Each panel (**A-C**) represents the average human-rated response quality for one question, with error bars indicating the standard deviation (SD) among 20 domain experts. The METABOLISM model consistently achieved the highest mean scores, indicating superior domain relevance and clarity compared with general-purpose models.

To complement the automated Phi-4 evaluation, a structured human expert assessment was conducted to validate the model’s interpretability and domain relevance. Twenty domain experts (each with research backgrounds in microbiology, biotechnology, nutrition, or systems biology) independently evaluated responses from three large language models (GPT-5, Gemini, and METABOLISM) across three microbiome-related prompts (Q1-Q3). Each response was scored on a five-point Likert scale (1 = poor, 5 = excellent) for overall quality, as shown in Fig. 5A-5C. Across all three questions, the METABOLISM model consistently received the highest mean ratings, followed by Gemini and GPT-5. Specifically, METABOLISM achieved average scores exceeding 4.5 ± 0.3 in all evaluations, whereas GPT-5 and Gemini exhibited broader variability (mean 2.5-4.2). The narrower standard deviation observed for METABOLISM indicates stronger inter-expert consensus, suggesting that domain adaptation effectively enhanced both clarity and contextual coherence. Notably, experts commented that responses from METABOLISM demonstrated higher biological plausibility, accurate interpretation of microbiome-host relationships, and reduced tendencies toward vague generalizations. Expert evaluations were designed to assess the biological plausibility and literature consistency of generated responses, rather than to validate biological accuracy or experimentally confirmed mechanisms. For example, when queried about gut microbiota–derived metabolites and liver inflammation, METABOLISM generated literature-consistent explanations linking short-chain fatty acids and bile acid signaling to hepatic metabolic regulation, reflecting established microbiome–liver axis mechanisms rather than novel biological claims. In this context, the model further articulated a plausible literature-based hypothesis suggesting that alterations in short-chain fatty acid–producing taxa may modulate hepatic inflammatory signaling through bile acid–mediated pathways, a relationship that has been repeatedly reported in experimental and clinical studies. These findings confirm that domain-specialized fine-tuning enables LLMs to generate context-aware scientific reasoning, particularly when integrating literature-derived evidence in microbiome research. The improvement in clarity and factual alignment is critical for practical applications such as hypothesis generation, experimental design, and literature curation—areas where conventional general-purpose LLMs often produce ambiguous or oversimplified outputs.

### Human Expert Rubric Outcomes and Safety Evaluation

**Fig. 6:**
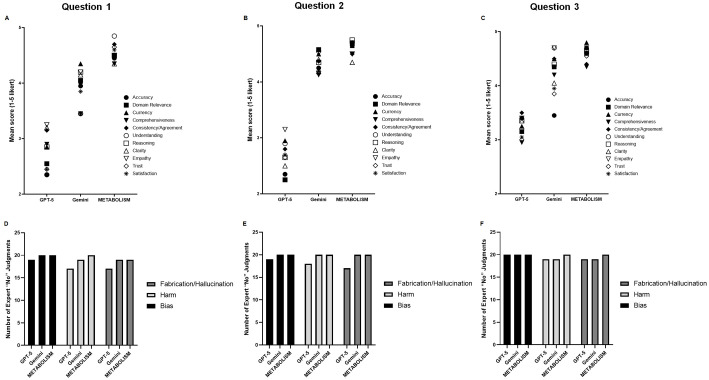
Human expert evaluation rubric outcomes for large language models in microbiome research. Twenty domain experts evaluated responses generated by three large language models (GPT-5, Gemini, and METABOLISM) to three microbiome-related questions (Q1-Q3) using a total of 14 rubric dimensions. Panels (**A-C**) show mean (±SD) expert ratings on 11 qualitative dimensions assessed with a five-point Likert scale (accuracy, domain relevance, clarity, usefulness, reasoning, etc.). Panels (**D-F**) present binary (Yes/No) evaluations of potential safety issues (fabrication/hallucination, harm, or bias) identified for each model.

To further dissect qualitative differences among models, 14 rubric dimensions were analyzed (Fig. 6). These covered accuracy, domain relevance, reasoning, clarity, comprehensiveness, usefulness, empathy, and trustworthiness. Again, METABOLISM demonstrated superior scores across nearly all dimensions, outperforming GPT-5 and Gemini in accuracy, domain relevance, and reasoning quality (mean > 4.5 for each) (Fig. 6A-6C). Particularly, the improvement in “domain relevance” and “clarity” suggests that continual exposure to microbiome-specific corpora during fine-tuning allowed the model to better capture the nuanced relationships among microbial taxa, metabolites, and host pathways. Panels D-F of Fig. 6 present the results of the binary safety evaluation, in which experts assessed the presence of potential issues such as fabrication/hallucination, harm, or bias. While minor instances of content fabrication were occasionally identified across all models, METABOLISM recorded the lowest frequency of hallucination-like responses and no harmful or biased statements (Fig. 6D-6F). This outcome highlights the potential of domain-adaptive learning to not only enhance precision but also reduce misinformation risk in biomedical AI applications.

Collectively, our results highlights the importance of human-centric benchmarking in the validation of LLMs for scientific use. Further, quantitative metrics such as BLEU or ROUGE alone may not be able to capture the depth of biological reasoning, factual consistency, or ethical safety considerations intrinsic to high-impact microbiome research. Integrating expert-guided rubrics into LLM evaluation thus provides a more reliable and interpretable framework for assessing trustworthiness and scientific utility.

## Supplemental Materials

Supplementary data for this paper are available on-line only at http://jmb.or.kr.



## Figures and Tables

**Fig. 7:**
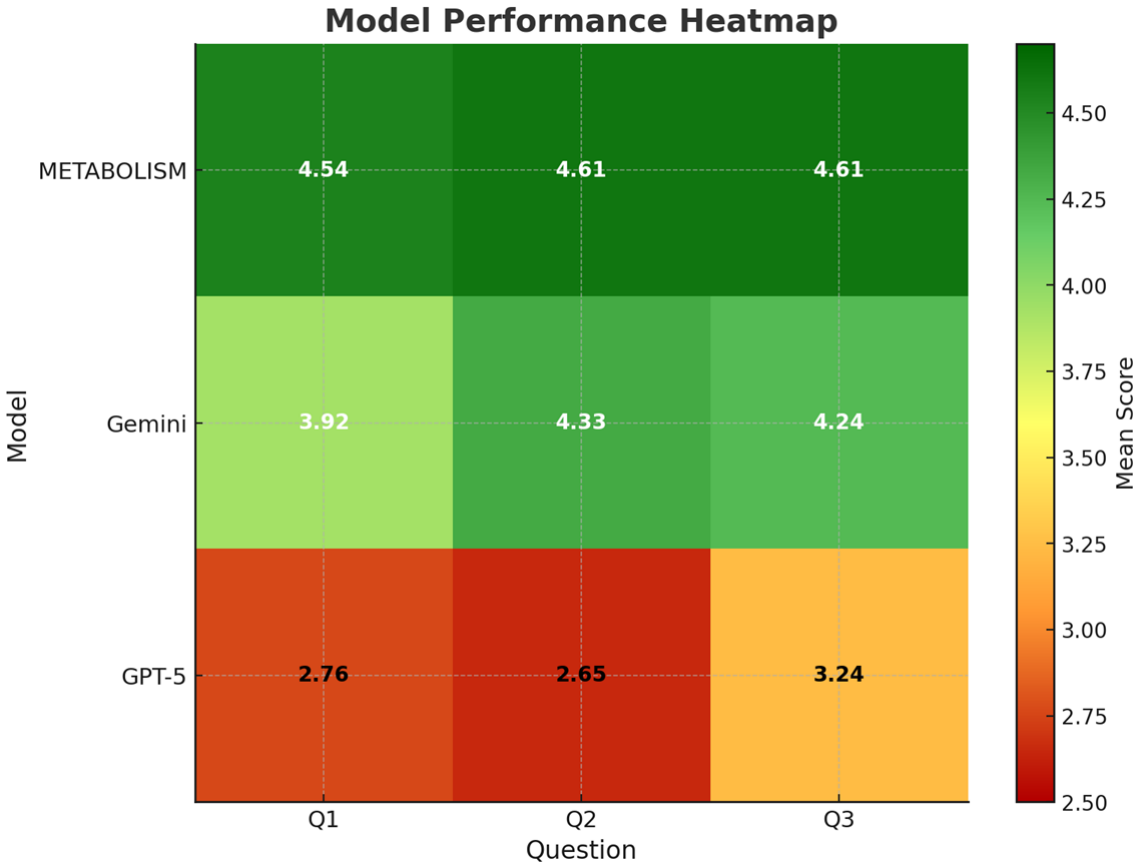
Model performance heatmap based on human expert evaluation. Mean expert ratings (1-5 Likert scale) for three large language models (GPT-5, Gemini, and METABOLISM) across three evaluation questions (Q1-Q3). Each cell represents the average score of 20 domain experts, with color intensity corresponding to the mean rating (red = low, gree*n* = high). The METABOLISM model achieved the highest scores across all questions, indicating superior domainspecific reasoning and response quality.
