# Supplementary material for: Development of Large Language Model Specialized into Microbiome Datasets: an Application of Self-Evaluation and Scoring Comparison with Conventional Natural Language Processing Markers
Source: J Microbiol Biotechnol. 2026 Jan 26;36:e2511050. doi: 10.4014/jmb.2511.11050 (PMC12868943; doi:10.4014/jmb.2511.11050)
Supplement: Supplementary file 1 [file jmb-36-e2511050-supple.pdf]

J. Microbiol. Biotechnol. 2026. 36: e2511050
https://doi.org/10.4014/jmb.2511.11050

Table 1. Rubric dimensions and definitions for human expert evaluation of large language model responses in microbiome research.

Category	Dimension	Definition
Quality of Information	Accuracy	Scientific correctness and factual validity of the content
Quality of Information	Domain Relevance	Specificity and appropriateness within the microbiome/life science context
Quality of Information	Currency	Reflects the most up-to-date knowledge and literature
Quality of Information	Comprehensiveness	Covers all critical aspects of the query without omissions
Quality of Information	Consistency/Agreement	Aligns with established knowledge; free of contradictions
Understanding & Reasoning	Understanding	Correct interpretation of the user's query and context
Understanding & Reasoning	Reasoning	Logical reasoning process and validity of supporting evidence
Expression & Persona	Clarity	Readability, coherence, and structural organization of the response
Expression & Persona	Empathy	Consideration of user perspective; avoids overly mechanical or insensitive tone
Safety & Harm Avoidance	Bias	Absence of systemic bias, prejudice, or discriminatory content
Safety & Harm Avoidance	Harm	Avoidance of misleading, harmful, or unsafe advice
Safety & Harm Avoidance	Fabrication/Hallucination	No made-up facts, unverifiable claims, or false references
Trust & Confidence	Trust	Overall reliability and credibility of the response
Trust & Confidence	Satisfaction	Overall evaluator satisfaction with the quality of the response

Table 2. Detailed human expert evaluation scores across 11 qualitative dimensions for three large language models.

Rubric Item	Dimension	LLM Type	Question 1		Question 2		Question 3	
			Mean	SD	Mean	SD	Mean	SD
Quality of Information	Accuracy	METABOLISM	4	0.92	4.65	0.49	4.7	0.47
		Gemini	2.35	0.98	4.25	0.72	3.45	0.94
		GPT-5	2.35	0.99	2.35	0.93	3.4	0.82
	Domain Relevance	METABOLISM	4.3	0.8	4.7	0.47	4.6	0.5
		Gemini	2.55	0.89	4.58	0.61	4.35	0.81
		GPT-5	2.55	0.89	2.25	0.85	3.15	0.99
	Currency	METABOLISM	4.4	0.75	4.65	0.49	4.8	0.41
		Gemini	3	1.17	4.5	0.61	4.5	0.83
		GPT-5	3.05	1.15	2.95	1	3.25	0.97
	Comprehensiveness	METABOLISM	4.25	0.85	4.5	0.51	4.35	0.59
		Gemini	3.25	1.12	4.12	0.81	4.2	0.89
		GPT-5	2.7	1.03	2.25	0.72	2.95	0.83
	Consistency/Agreement	METABOLISM	4.65	0.48	4.5	0.51	4.4	0.5
		Gemini	3.2	0.92	4.37	0.68	4.5	0.89
		GPT-5	3.2	0.92	2.8	0.77	3.5	0.95
Understanding & Reasoning	Understanding	METABOLISM	4.85	0.36	4.7	0.47	4.7	0.73
		Gemini	4.45	0.6	4.55	0.6	4.6	0.5
		GPT-5	4.85	0.36	2.9	0.97	3.2	0.89
	Reasoning	METABOLISM	4.5	0.51	4.75	0.44	4.65	0.49
		Gemini	4.5	0.51	4.35	0.67	4.4	0.88
		GPT-5	4.5	0.51	2.65	0.88	3.35	0.88
Expression Style & Persona	Clarity	METABOLISM	4.35	0.49	4.35	0.59	4.7	0.57
		Gemini	4.45	0.51	4.4	0.5	4.05	0.69
		GPT-5	4.35	0.49	2.5	0.76	3.35	0.81
	Empathy	METABOLISM	4.5	0.51	4.5	0.51	4.7	0.47
		Gemini	4.5	0.51	4.15	0.75	4.7	0.47
		GPT-5	4.5	0.51	3.15	0.81	3.4	0.5

**Trust & Confidence**	**Trust**	**METABOLISM**	**4.5**	**0.51**	**4.7**	**0.47**	**4.55**	**0.51**
		**Gemini**	**4**	**0.79**	**4.15**	**0.88**	**3.85**	**0.99**
		**GPT-5**	**2.9**	**1.02**	**2.65**	**0.88**	**3**	**0.86**
	**Satisfaction**	**METABOLISM**	**4.6**	**0.5**	**4.65**	**0.49**	**4.6**	**0.5**
		**Gemini**	**4.1**	**0.72**	**4.2**	**0.7**	**3.95**	**1.1**
		**GPT-5**	**2.95**	**1**	**2.7**	**0.86**	**3.05**	**0.94**
